# Fine Mapping the Soybean Mosaic Virus Resistance Gene in Soybean Cultivar Heinong 84 and Development of CAPS Markers for Rapid Identification

**DOI:** 10.3390/v14112533

**Published:** 2022-11-16

**Authors:** Yong Li, Xinlei Liu, Wenjia Deng, Jiahui Liu, Yue Fang, Ye Liu, Tingshuai Ma, Ying Zhang, Yongguo Xue, Xiaofei Tang, Dan Cao, Zhifei Zhu, Xiaoyan Luan, Xiaofei Cheng

**Affiliations:** 1 School of Life Science, Northeast Agricultural University, Harbin 150030, China; 2 Soybean Research Institute, Heilongjiang Academy of Agricultural Science, Harbin 150086, China; 3 Key Laboratory of Germplasm Enhancement, Physiology and Ecology of Food Crops in Cold Region of Chinese Education Ministry, College of Agriculture, Northeast Agricultural University, Harbin 150030, China

**Keywords:** soybean mosaic virus, bulk segregation analysis (BSA), resistance locus, cleaved amplified polymorphic sequences (CAPS) markers, *Rsv1*

## Abstract

Heinong 84 is one of the major soybean varieties growing in Northeast China, and is resistant to the infection of all strains of soybean mosaic virus (SMV) in the region including the most prevalent strain, N3. However, the resistance gene(s) in Heinong 84 and the resistant mechanism are still elusive. In this study, genetic and next-generation sequencing (NGS)-based bulk segregation analysis (BSA) were performed to map the resistance gene using a segregation population from the cross of Heinong 84 and a susceptible cultivar to strain N3, Zhonghuang 13. Results show that the resistance of Heinong 84 is controlled by a dominant gene on chromosome 13. Further analyses suggest that the resistance gene in Heinong 84 is probably an allele of *Rsv1*. Finally, two pairs of single-nucleotide-polymorphism (SNP)-based primers that are tightly cosegregated with the resistance gene were designed for rapidly identifying resistant progenies in breeding via the cleaved amplified polymorphic sequence (CAPS) assay.

## 1. Introduction

Soybean (*Glycine max* (L.) Merr.) is one of the most important economic crops worldwide, providing high-quality vegetable oil and protein for human and livestock. The infection of phytopathogens, including plant viruses, can cause significant losses to soybean crops in both yield and quality [1]. Soybean mosaic virus (SMV) is one of the most economically important pathogens threating soybean production worldwide, and typically causes 8–35% yield loss, but may reach up to 50–100% losses in high-incidence fields [2]. SMV is a member of the genus *Potyvirus* in the family *Potyviridae* [3]. The genome of SMV comprises a single-stranded, positive-sense RNA molecule of about 9600 nt in length, which contains only one large open reading frame (ORF) that is translated into a large polypeptide. In addition, a unique polymerase slippage motif within the *P3* cistron enables the expression of an additional short polypeptide [4]. The two polypeptides are proteolytically processed by three viral proteases into 11 mature proteins, namely, protein 1 (P1), helper-component proteinase (HcPro), protein 3 (P3), P3 N-terminal fused with a pretty interesting potyviridae ORF (P3N-PIPO), 6-kilodalton protein 1 (6K1), cylindrical inclusion (CI), 6-kilodalton protein 2 (6K2), viral genome-linked protein (VPg), nuclear inclusion a-proteinase (NIa-Pro), nuclear inclusion b (NIb), and coat protein (CP) [5].

The symptoms caused by SMV vary depending on the virus isolate and soybean variety, including leaf mosaic, mottling, wrinkling, stem necrosis, stunting, and dwarfing [3]. According to the symptoms of SMV on a set of susceptible and resistant soybean cultivars, SMV was divided into seven strains (G1–G7) in the United States [6]. In Japan, SMV is classified into five strains (A–E) based on both biological and sequence properties [7]. China is believed to be the origin center of the bean common mosaic virus lineage of potyviruses including SMV [8]. Indeed, analyses suggest that the genetic diversity of SMV is very abundant in China [9]. Based on a set of different susceptible and resistant soybean cultivars in China, SMV is classified into at least 22 strains (SC1–SC22) of five genetic clusters (I–V) [10]. In Northeast China, three additional local strains of SMV (N1–N3) were characterized with N3 as the most pathogenic and prevalent [11,12]. Phylogenesis and symptomology analyses suggest that the genomes of SMV N1 and N3 are closely related and are clustered with SC3 and SC6 in the same phylogenetic clade, but their pathogenicity is most closely related to SC18 [9,12].

It is well-known that utilization of resistant cultivars is the most economic means to manage SMV. Hitherto, across more than a decade, resistance genes against SMV have been identified and mapped to chromosomes 2, 6, 13, and 14 [3,13]. However, only three SMV resistant genes have been cloned thus far: *Rsv4* on chromosome 2 encodes a RNase H family protein with substrate specificity on double-stranded RNA that is able to confer resistance to all SMV isolates [14]; *Rsc4-3* on chromosome 14 of Dabaima encodes a cell-wall-located nucleotide-binding domain leucine-rich protein (NLR) by recognizing the CI protein [15]; *Rsvg2* on chromosome 13 encodes a sulfotransferase (SOT) [16]. Heinong 84 is one of the main soybean varieties in the Northeast China, and is resistant to both SMV strains N1 and N3, most strains of *Cercospora sojina*, and all known strains of soybean cyst nematode [17]. The SMV resistance gene in Heinong 84 is derived from Ha91R3-301, an old local soybean variety [18]. However, the genetic information of the SMV resistance gene in Heinong 84 is elusive, which hampers the utilization of this gene in genetic breeding. In this study, we report the mapping of the SMV resistance gene in Heinong 84 with a segregation population from the cross of Heinong 84 and a susceptible cultivar Zhonghuang 13 by the next-generation-sequencing (NGS)-based bulk segregation analysis (BSA), a gene mapping method widely used to localize quantitative trait loci (QTL) [19,20]. Moreover, the candidate resistant genes were analyzed, and several cleaved amplified polymorphic sequence (CAPS) markers were developed for rapid identification.

## 2. Materials and Methods

### 2.1. Soybean Materials and Growth Conditions

Soybean cultivars Heinong 84 and Zhonghuang 13 have been characterized previously [17,21]. Heinong 84 (resistant male parent) and Zhonghuang 13 (susceptible female parent) were crossed at the experimental station of the Soybean Research Institute of Heilongjiang Academy of Agricultural Science in the summer of 2018. Zhonghuang 13 instead of Williams 82 was selected as the female parent since the genome of the former has been sequenced at extremely high quality, which will benefit the subsequent BSA [21,22]. A small number of F_1_ seeds were inoculated with SMV N3 for resistance analyses and the rest of the F_1_ individuals were self-pollinated to produce the segregating F_2_ population. In the summer of 2020, a total number of 593 F_2_ individuals were inoculated by SMV strain N3 and their resistance was evaluated; the rest of the non-inoculated F_2_ progenies were individually harvested to form the segregating F_3_ population. In 2021, a total number of 643 F_3_ progenies were planted, inoculated by SMV strain N3, and their resistance was recorded; leaf samples were collected for NGS-based BSA. The chi-square (χ^2^) test was used to evaluate the fit of observed to expected segregation ratios in all populations.

### 2.2. Virus Resource and Mechanical Inoculation

SMV strain N3 was provided by the Soybean Research Institute of the Northeast Agricultural University and was maintained on susceptible soybean cultivar Dongnong 50 or Hefeng 25 in an insect-proof greenhouse [11]. The inoculum was prepared by homogenizing the leaf tissues of Dongnong 50 that were infected by SMV N3 in 0.01 mol/L phosphate buffer (pH 7.2). Mechanical inoculation was performed as described previously [23]. In brief, the unifoliate leaves of 10- to 12-day-old soybean seedlings were dusted by 600-mesh carborundum powder and gently rubbed with the viral inoculum 3–4 times. After leaving this on for 2–3 min, the inoculated leaves were rinsed with distilled water and covered with a pre-wetted paper towel. Inoculated plants were returned to the greenhouse for symptom development. Symptoms were recorded twice a week from 7 days post-inoculation (dpi) for at least three weeks. The phenotypes were recorded based on a 5-grade disease index [24]: grade 0, no symptom; grade 1, mild mosaic with no shrinkage or curling; grade 2, mild mosaic with shrinkage or curling and slight dwarf; grade 3, severe mosaic with significant shrinkage or curling, necrotic spots, vein necrosis, and moderate dwarf; grade 4, severe mosaic symptoms, severe dwarf or stem-tip necrosis. Necrotic plants were classified as the resistant group as they contain resistance genes regardless of the symptom expression [25].

### 2.3. Enzyme-Linked Immunosorbent Assay (ELISA)

ELISA was performed using the ELISA reagent set for soybean mosaic virus (SMV) (Agdia, Elkhart, IN, USA) according to manufacturer’s protocol with triple technical repeats. The optical density reads at 450 nm (OD_450_ value) were recorded using a Varioskan MUTIPLATE plate reader (Thermo Fisher Scientific, Shanghai, China). For each sample, the OD_450_ value was subtracted from the value of the blank control (no leaf tissue) and then compared to the value of the healthy control (healthy soybean leaf tissue).

### 2.4. DNA Extraction, Library Preparation, and High-Throughput Sequencing

Genomic DNA was extracted from the soybean leaves using the Fastpure plant DNA isolation mini kit (Vazyme, Nanjing, Jiangsu, China) according to the manufacturer’s protocol. The concentration and quality of genomic DNA samples were determined by a NanoDrop 2000 microvolume spectrophotometer (Thermal Fisher Scientific). The quality of each DNA sample was further assessed by electrophoresis on 0.8% agarose gel. Then, the resistant and susceptible pools were generated by pooling equal amounts of DNA from 50 resistant and 50 susceptible individuals, respectively. About 5 μg of DNA from the two pools or two parental lines was used to construct paired-end sequencing libraries, which were sequenced on an Illumina NovaSeq^TM^ 6000 platform at Hangzhou Lianchuan Biotechnology Co., Ltd. (Hangzhou, China).

### 2.5. Bulk Segregation Analysis

The adaptor sequence on reads from a HiSeqTM 2500 platform were trimmed, and low-quality reads were discarded using a homemade python script. The resulting high-quality reads were mapped to the reference genome of Zhonghuang 13 (DDBJ/ENA/GenBank accession QKRT00000000) using HiSat2 v2.2.1 with parameter end-to-end [26]. Single-nucleotide polymorphism (SNP) calling was carried out after removing duplication using samtools v1.15 [27]. QTLseqr (v0.7.5.2) was used to localize resistant loci (SNPs with a sample depth less than 15 or total depth less than 40 were removed) [28].

### 2.6. Cleaved Amplified Polymorphic Sequences (CAPS) Primer Design and Validation

According to the mapping results generated by HiSat2, SNPs in the target gene locus were identified using bedtools v2.30 [29]. SNP2CAPS v0.6 was applied to convert SNPs to CAPS markers [30]. CAPS primers were designed at both sides of the SNP-containing sites by Primer Premier v5.0 with default settings (Table 1). Polymerase chain reactions (PCR) were performed in a 20 μL volume system containing 10 μL of 2 × Rapid Taq master mix (Vazyme), 0.5 μL each of forward and reverse primers (10 mmol/L), 0.5 μL of template DNA (100 ng/μL), and 8.5 μL of sterile water. PCR was performed using a T30D tri-block super-gradient PCR system (LongGene, Hangzhou, Zhejiang, China) with the following PCR program: predenaturation at 95 °C for 3 min, followed by 30 cycles of denaturation at 95 °C for 15 s, annealing at the primer melting temperature (Tm) for 30 s, extension at 72 °C for 1 s, and a final extension at 72 °C for 10 min. PCR products were digested with restriction enzyme *Xba* I (New England Biolabs, Beijing, China) in a 25 μL volume system containing 2.5 μL of 10 × CutSmart buffer, 0.5 μL of *Xba* I, 10 μL of PCR product, and 9 μL ddH_2_O. The mixture was incubated at 37 °C for 30 min, and 65 °C for 20 min to inactivate the enzyme, and then analyzed by electrophoresis on a 1.8% agarose gel. The amplified bands on each photo were analyzed by imageJ and statistical analyses were performed using the Student’s *t*-test.

### 2.7. Sequence Clone, Alignment, and Comparison

The amino acid sequences of SMV strains G7, G7d, and N were retrieved from Genbank under the accession nos. AY216010, AY216987, and D00507, respectively. The *HcPro* and *P3* of SMV N3 were amplified from total RNA extracted from leaves of Dongnong 50 that infected by SMV N3 using the Phanta max super-fidelity DNA polymerase (Vazyme) with the primers shown in Table 1. The amplified fragments were ligated into pCE2-TA-Blunt-Zero vector (Vazyme) and Sanger sequenced at RuiBiotech Co., Ltd. (Beijing, China). Amino acid sequences were aligned with Clustal Omega with default settings [31].

## 3. Results

### 3.1. Genetic Analysis of the Resistance of Heinong 84 to SMV N3

**Figure 1:**
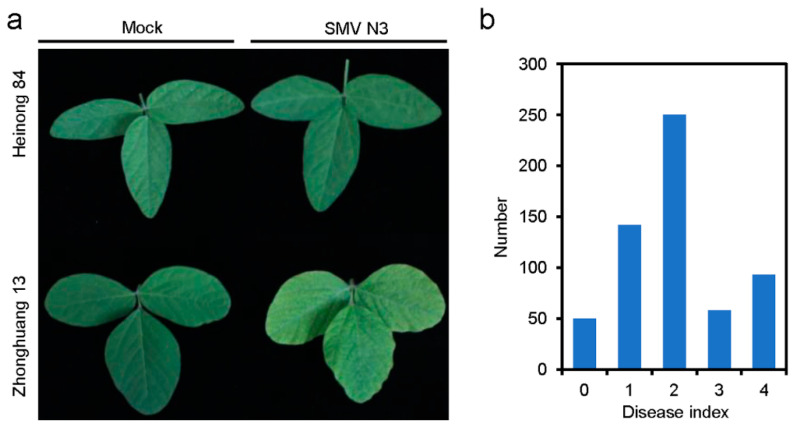
Resistance of Heinong 84, Zhonghuang 13, and their hybrid F_2_ offspring. (**a**) Phenotypes of systemic leaves of Heinong 84 and Zhonghuang 13 inoculated by buffer (mock) or SMV N3 at 20 dpi. (**b**) Bar chart showing the distribution of the F_2_ populations in different disease indices.

Three-week-old seedlings of Heinong 84 and Zhonghuang 13 were mechanically inoculated by SMV N3 on the first trifoliate compound leaf. The upper non-inoculated leaves of all Zhonghuang 13 seedlings (n = 5) displayed typical SMV symptoms including leaf mosaic and wrinkling as early as 10 days post-inoculation (dpi), while no visible lesions from hypersensitive responses were observed on the inoculated leaves of Heinong 84 (n = 5) and no obvious viral symptom was observed on upper non-inoculated leaves of Heinong 84 throughout the whole experiment period (Figure 1a), confirming the resistance and susceptibility of Heinong 84 and Zhonghuang 13 to SMV N3, respectively. To determine the resistance of Heinong 84, we crossed Heinong 84 and Zhonghuang 13. All seedlings of the F_1_ generation showed resistance to SMV N3, indicating the resistance is dominantly inherited. Seedlings of the F_1_ generation were self-pollinated, and the resistance in the F_2_ generation was further evaluated. The results showed that 442 out of 593 (74.5%) F_2_ seedlings were resistant (disease index grade 0–2), and the other 151 (25.5%) plants were susceptible (disease index grade 3–4; Figure 1b; Table 2). The segregation ratio matched 3:1 (χ^2^ = 0.667; *p* = 0.414; Table 2), indicating that the resistance of Heinong 84 is controlled by a dominant gene.

### 3.2. BSA Pool Preparation and Sequencing

**Figure 2:**
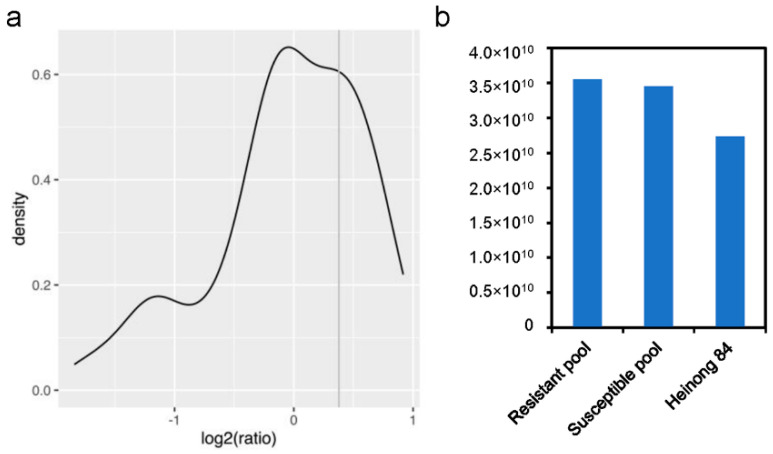
Characterization of the resistant and susceptible populations. (**a**) Distribution of the OD_450_ values of the resistant pool. (**b**) Bar chart showing the number of bases with quality higher than 30 of the resistant pool, susceptible pool, and Heinong 84 from the NGS.

To exclude potential false-resistant individuals, each of the seedlings with a disease index grade 0 or 1 was further evaluated by ELISA (Table S1). Statistical analyses showed that when the ratio of the OD_450_ value of the sample to that of the healthy control is <1.3, they can be recognized as extremely resistant individuals (Figure 2a). Based on this criterion, the DNA of 50 extremely resistant seedlings was selected and equally mixed as the resistant pool, while the susceptible pool consisted of 50 samples with disease index grade 4. The susceptible and resistant libraries together with their parent Heinong 84 were sequenced using the Illumina platform. After adaptor trimming and quality control, a total number of 35.5, 34.5, and 27.3 trillion high-quality data (quality score ≥ 30) of the resistant pool, susceptible pool, and Heinong 84 were obtained, respectively (Figure 2b). These data were used for the subsequent BSA.

### 3.3. Mapping the Resistance Locus in Heinong 84

**Figure 3:**
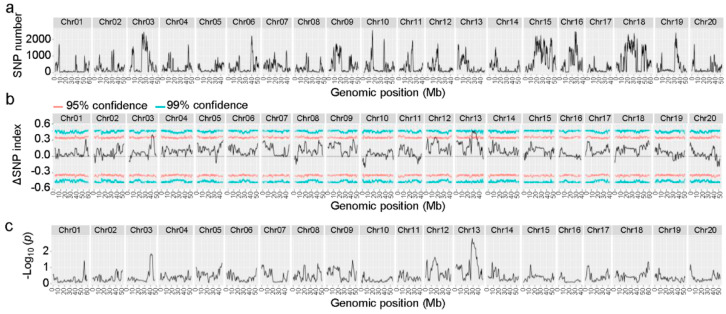
Distribution and plot of SNPs. (**a**) Distribution of all SNPs on twenty soybean chromosomes. (**b**) ΔSNP index graphs from NGS-based BSA. (**c**) Distribution of p value (presented as -log_10_). X-axis represents the position of twenty chromosomes and Y-axis represents the number of SNPs (**a**), ΔSNP index (**b**), or *p* value (**c**). ΔSNP index was calculated in a 1 Mb interval with a 10 kb sliding window. The red and cyan lines indicate expectation values at 95% confidence (*p* < 0.05) and 99% confidence (*p* < 0.01), respectively, under the null hypothesis.

The resulting clean reads were mapped to the genome assembly of Zhonghuang 13. SNPs were retrieved, filtered, and classified. A total number of 393,979 SNPs that were distributed on all 20 chromosomes were used for subsequent BSA to locate the N3 resistance locus. At 95% confidence, three genomic regions were identified as putative resistant quantitative trait loci (QTLs) (Figure 3). The first resistant locus was located on chromosome 3 (39,435,027–43,157,802 bp), being about 3.72 Mb in size, the second resistant QTL locus was located on chromosome 12 (15,669,309–16,956,035 bp), being about 1.29 Mb in size, and the third QTL locus was located on chromosome 13 (27,358,662–35,308,006 bp), being about 7.95 Mb in size. Among all known SMV resistance genes or loci, only *Rsv1* and its alleles are located on chromosome 13, while no SMV resistance gene or locus has been mapped to chromosomes 3 or 12 [3]. Moreover, only the locus on chromosome 13 reached the 99% confidence interval (Figure 3b,c), indicating that the SMV resistance gene in Heinong 84 is most probably located in this region. Since the genetic analysis suggests the resistance in Heinong 84 is controlled by a dominant gene, we focused our study in the locus on chromosome 13, and this is referred to as *R_SMV-N3_* hereafter.

### 3.4. Resistant Gene Prediction

Based on the gene annotation of Zhonghuang 13, a total number of 795 genes were found in the resistant locus of chromosome 13, among which 557 genes were found to be different between Heinong 84 and Zhonghuang 13. Based on the location (untranslated region, coding region, intron, etc.) and effect (synonymous, mis-sense, nonsense, etc.) of SNPs, the 557 genes were further classified into two groups, namely high-impact and low-impact groups. The first group contained 51 genes, which have SNPs/indels that caused frameshift, early translation termination, or gained additional amino acids by intron retain or stop codon lost (Table S2); the second group contained genes with intron variant, UTR variants, synonymous variants, or mis-sense variant. Interestingly, gene function annotation showed that 5 of the 51 genes encode nucleotide-binding domain leucine-rich repeat (NLR) proteins (Table S2), indicating that *R_SMV-N3_* may also encode an NLR protein.

**Figure 4:**
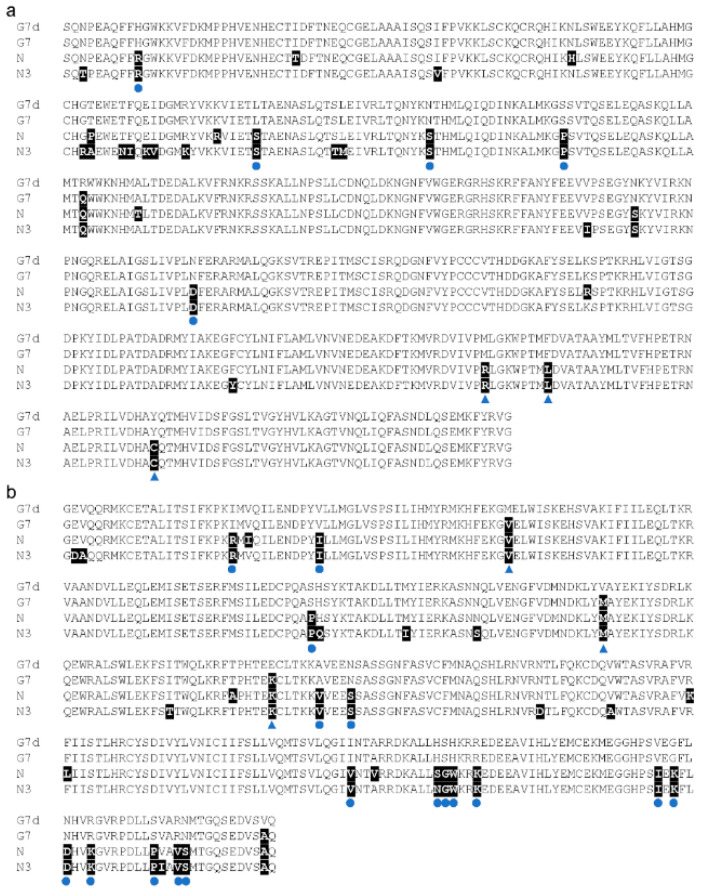
Multiple alignment of the partial amino acid sequences of SMV N3, N, G7, and G7d. (**a**) HcPro. (**b**) P3. Varied residues are highlighted in black, residues that are involved in the evading *Rsv1*-mediated resistance have been indicated by blue triangles, and residues consistent in N and N3 are indicated by blue dots.

Studies have shown that *Rsv1* and its alleles encode NLR proteins that confer an extreme resistance [32], a type of effector-triggered immunity (ETI), without a hypersensitive response [33]. SMV N3 also could not induce HR on the inoculated leaves of Heinong 84, indicating that *R_SMV-N3_* may also encode an NLR protein. Since the elicitors of *Rsv1* (P3 and HcPro) have been well-documented [34,35], we directly compared the sequence variations of P3 and HcPro between SMV strains N3, N, G7, and G7d. N is an avirulent strain on *Rsv1*-carring cultivars, e.g., PI 96983, and G7 is a virulent strain that can provoke a lethal systemic hypersensitive response (LSHR), while G7d is an experimentally evolved variant of G7 that can completely evade the *Rsv1*-mediated resistance [36]. The nucleotide of HcPro and P3 of SMV N3 were cloned, and their deduced amino acid sequences were compared with that of SMV strains N, G7, and G7d. Results show that all residues required for G7 and G7d to evade *Rsv1*-mediated resistance were not found in the amino acid sequences of HcPro and P3 of SMV N3 (Figure 4), indicating that *R_SMV-N3_* may be an allele of *Rsv1*.

### 3.5. Development of CAPS Marker for Rapid Identification

**Figure 5:**
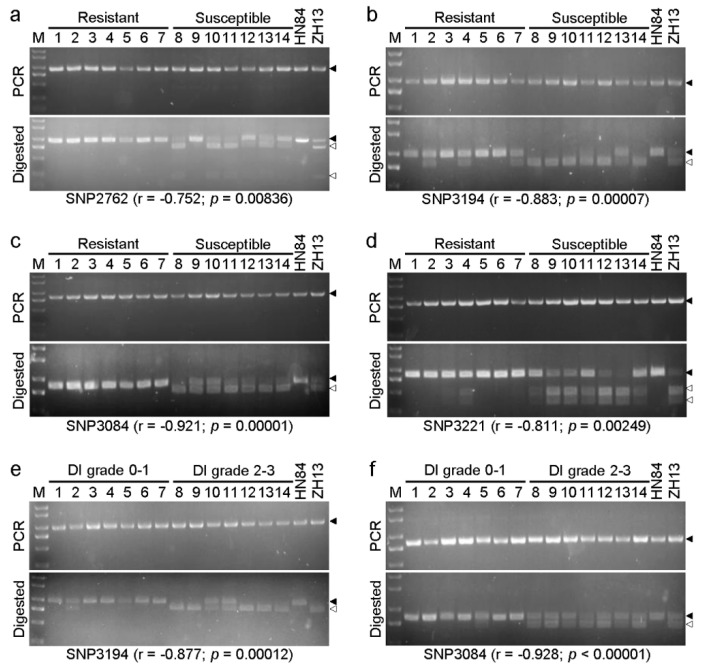
CAPS assays. (**a**–**d**) Restriction enzyme digestion and gel electrophoresis of PCR products with primer pair SNP2762 (**a**), SNP3194 (**b**), SNP3084 (**c**), and SNP3221 (**d**). M, DL2000 Plus DNA marker (Vazyme Biotech); lanes 1–7, resistant DNA pool (seven samples per lane); lanes 8–14, susceptible pool (seven samples per lane). (**e**,**f**) CAPS assays of seven resistant or susceptible individuals using the primers based on SNP3194 (**e**) and SNP3084 (**f**); lanes 1-14 represent 14 random-selected samples; DI grade, disease index grade. HN84 and ZH13 represent total DNA of Heinong 84 and Zhonghuang 13, respectively. The solid and hollow arrow heads indicate amplified fragments and digested fragments, respectively. Note that some *Xba* I-digested fragments may not be visible on the agarose gel due to small size. Statistical analysis was performed using the Student’s *t*-test. Pearson correlation coefficients (r) are also indicated.

The CAPS-marker-based assay is a simple, cheap, and reliable method for detecting SNP variation, which is particularly useful in crop breeding. Four pairs of primers were designed and synthesized based on the SNPs in the *R_SMV-N3_* locus of chromosome 13 for rapid identification of the resistant progenies of Heinong 84 (Table 1). PCR amplification, restriction enzyme digestion, and subsequent gel electrophoresis showed that all four pairs of primers can distinguish between the two parents (Figure 5a–d), indicating that these SNP are indeed present in the two parents. We then analyzed the reliability of these primers using the resistant and susceptible libraries. The total DNA of seven individuals of the resistant or susceptible pool were mixed as a biological repeat to reduce the number of PCR reactions. Results show that all CAPS primers could be used for separating the resistant and susceptible pools (Pearson correlation coefficient r ≥ −0.752; *p* ≤ 0.00836) with SNP3084-based primers having the highest Pearson correlation coefficient (r = −0.921; *p* = 0.00001) (Figure 5a–d). These results show that these SNPs are tightly cosegregated with *R_SMV-N3_* with SNP3084 in the closest proximity to the resistance locus. We further analyzed seven randomly selected resistant (disease grade 0 or 1) or susceptible (disease grades 2 or 3) individuals of the F_3_ generation using the SNP3194- and SNP3084-based primers. Results show that both pairs of primers successfully detected all susceptible and six of the seven resistant samples (r ≥ −0.877; *p* ≤ 0.00012) (Figure 5e).

## 4. Discussion

SMV is the most economically important viral pathogen of soybean worldwide. Identification of the resistant gene will certainly benefit the breeding of resistant soybean cultivars. In this study, we tried to localize the SMV resistance locus in Heinong 84 using a separation population crossed from Heinong 84 and the susceptible cultivar Zhonghuang 13. Genetic analysis suggested that the resistance of Heinong 84 to SMV N3 is most probably controlled by a dominant gene (Table 2). Previous studies have confirmed that the resistant gene in Heinong 84 is derived from Ha90-33-2 [17]. Genetic analyses on several hybrid populations that were crossed from Ha91R3-301 and susceptible cultivars such as Kennong 4, Ha90-33-2, and Heinong 41 showed that Ha91R3-301 has two dominant but complementary resistant genes to SMV N3 [18,37]. These results suggest that one resistance gene may be lost during the breeding of Heinong 84. Interestingly, BSA identified three putative resistant QTL loci on chromosome 3, 12, and 13 in Heinong 84 with the loci on chromosome 13 having the highest confidence (Figure 3). These results suggest that besides the dominant gene, there may be one or two recessive or additive genes contributing to the resistance to SMV N3 in Heinong 84.

Our results show that the location of the major resistant QTL locus on chromosome 13, which is the same gene locus of *Rsv1* from PI96983 and its alleles, such as *Rsc-ps* and *Rsc-pm* from PI96983, *Rsv1-h* from Suweon97, *Rsc3Q* and *Rsc14Q*/*Rsc14* from Qihuang 1, *Rsc12* and *Rsc18Q* from Qihuang 22, *Rsv1-y* from York, *Rsv1-t* from Ogden, and *Rsv1-m* from Marshall [3,6,25]. The resistant QTL locus on chromosome 13 (F locus) is highly complex as it contains a cluster of NLR genes [38]. SMV P3 has been identified as the elicitor of *Rsv1*-mediated resistance [34]. However, the gain of virulence by an avirulent SMV strain, e.g., strain N, on *Rsv1*-genotype soybean (PI96983), requires concurrent mutations in both the HcPro and P3 [35], indicating there may be more than one resistant gene in the F locus of PI96983. Indeed, two distinct NLR proteins that mediate recognition of the C-terminal of HcPro and the N-terminal 271 amino acids of P3 respectively were identified within the F locus using two soybean lines that derived from crosses between PI96983 and Lee68 (*Rsv1*) with distinct recombination events inside the locus [35,39]. No necrotic spots (hypersensitive responses) were observed on the leaves of Heinong 84 that were mechanically inoculated by SMV N3, and all the three key residues that are required for escaping the *Rsv1* recognition in the P3 of SMV N3 are identical to that of the avirulent SMV N strain (Figure 4), indicating that *R_SMV-N3_* is also an allele of *Rsv1* and also recognizes the P3 and/or HcPro of SMV N3, and Heinong84 may have a similar resistance spectrum to PI96983. Nevertheless, further investigations are needed to fully uncover these possibilities.

Lots of locus markers, e.g., BARCSOYSSR_13_1114, BARCSOYSSR_13_1115, BARCSOYSSR_13_1140, BARCSOYSSR_13_1155, BARCSOYSSR_13_1128, BARCSOYSSR_13_111436, Satt334, Sct_033, Satt234, and SOYHSP176, have been located in the *Rsv1* locus of chromosome 13 [3]. However, the applicability and actual distance of these markers to the *R_SMV-N3_* in Heinong 84 are unknown. To overcome this deficiency, a set of CAPS markers have been developed for quick screening of resistant progenies of Heinong 84. Our results show that all four pairs of primers are cosegregated with *R_SMV-N3_*. Of note, the primers based on SNP3194 and SNP3084 were able to distinguish almost all susceptible and six out of seven randomly selected resistant individuals (Figure 5). Given the fact that the amplicon from heterozygous variants will be partially digested and recombination may take place between the SNP and resistant gene, the CAPS assay does not detect all resistant variants. Nevertheless, SNP-based primers have a higher cosegregation ratio with the resistance gene compared with genetic markers. Thus, our results indicate that primers based on SNP3194 and SNP3084 are in close proximity to the *R_SMV-N3_* locus on chromosome 13 and can be utilized in future breeding practice.

## 5. Conclusions

In conclusion, a segregation population was produced by crossing resistant cultivar Heinong 84 and susceptible Zhonghuang 13. The dominant resistance gene in Heinong 84 to SMV N3 (*R_SMV-N3_*) was mapped to chromosome 13 by NGS-based BSA. Two pairs of CAPS markers that are highly co-separated with *R_SMV-N3_* were designed for rapid identification of resistant progenies for future breeding.

## Figures and Tables

**Table 1:** Primers used in the present study.

Primer Names	Sequences (5′–3′)	Usage
N3-HcPro_F	TCCCAAACTCCTGAAGCTCAA	Cloning
N3-HcPro_R	ACCAACTCTATAAAATTTCATCTC	Cloning
N3-P3_F	GGTGATGCGCAACAAAGGATG	Cloning
N3-P3_R	CTGTGCGGAAACATCTTCTGATTG	Cloning
SNP2762_F	TAGTGGTGGATGGTTATGC	CAPS assay
SNP2762_R	TTTCCTGGCTGTTCCTATT	CAPS assay
SNP2805_F	TGAAAGTGGCTATGCTAT	CAPS assay
SNP2805_R	AATCAACCCTCCAAATCG	CAPS assay
SNP3084_F	CAACTGTATGGTTTAGGGATT	CAPS assay
SNP3084_R	AATTAGAGTGACCTGCAAGAT	CAPS assay
SNP3194_F	TTCTCCTACGGTCATTGTT	CAPS assay
SNP3194_R	TTTCTTATGTATGCTGGTG	CAPS assay
SNP3221_F	TCAATGACCCTTTGTGAG	CAPS assay
SNP3221_R	GGGAGGCTTGTCTACTGC	CAPS assay

**Table 2:** Genetic analyses of the resistance of the two parents and their hybrid populations to SMV N3.

Parents and Offspring	Resistant	Susceptible	Total	Theoretical Separation Ratio	χ^2^	*p*
Heinong 84	14	0	14			
Zhonghuang 13	0	14	14			
F_1_	12	0	12			
F_2_	442	151	593	3:1	0.667	0.414

## Data Availability

All data have been included in the text or as supplementary files.

## Supplementary Materials

The following supporting information can be downloaded at: https://www.mdpi.com/article/10.3390/v14112533/s1, Table S1: ELISA reads of the resistant pool; Table S2: The list of high-impact candidates in Heinong 84.
